# Magellan: A Web Based System for the Integrated Analysis of Heterogeneous Biological Data and Annotations; Application to DNA Copy Number and Expression Data in Ovarian Cancer

**Published:** 2007-02-05

**Authors:** Chris B. Kingsley, Wen-Lin Kuo, Daniel Polikoff, Andy Berchuck, Joe W. Gray, Ajay N. Jain

**Affiliations:** 1 UCSF Cancer Research Institute and Comprehensive Cancer Center, University of California, San Francisco, 2340 Sutter St., San Francisco California, USA.; 2 Department of Laboratory Medicine and UCSF Comprehensive Cancer Center, University of California San Francisco, San Francisco, California,; 3 Department of Obstetrics and Gynecology, Division of Gynecologic Oncology, Duke University Medical Center, Durham, North Carolina, USA.

**Keywords:** Online Data Analysis, Biological Annotations, Ovarian Cancer

## Abstract

Recent advances in high throughput biological methods allow researchers to generate enormous amounts of data from a single experiment. In order to extract meaningful conclusions from this tidal wave of data, it will be necessary to develop analytical methods of sufficient power and utility. It is particularly important that biologists themselves be able to perform many of these analyses, such that their background knowledge of the experimental system under study can be used to interpret results and direct further inquiries. We have developed a web-based system, Magellan, which allows the upload, storage, and analysis of multivariate data and textual or numerical annotations. Data and annotations are treated as abstract entities, to maximize the different types of information the system can store and analyze. Annotations can be used in analyses/visualizations, as a means of subsetting data to reduce dimensionality, or as a means of projecting variables from one data type or data set to another. Analytical methods are deployed within Magellan such that new functionalities can be added in a straightforward fashion. Using Magellan, we performed an integrated analysis of genome-wide comparative genomic hybridization (CGH), mRNA expression, and clinical data from ovarian tumors. Analyses included the use of permutation-based methods to identify genes whose mRNA expression levels correlated with patient survival, a nearest neighbor classifier to predict patient survival from CGH data, and curated annotations such as genomic position and derived annotations such as statistical computations to explore the quantitative relationship between CGH and mRNA expression data.

## Background

Recent advances in high-throughput genomic and proteomic analysis are generating enormous amounts of quantitative biological data. While there are distinct advantages to comprehensively quantifying biological variables such as mRNA expression level or genomic copy number across many genes and genomic loci, the scale of the data presents statistical challenges. The ratio of the number of measurements to the number of samples can become very large, which can result in spurious statistical observations. Many groups have addressed this ‘multiple comparisons problem’. Resampling and permutation based approaches, for example, can help to establish meaningful significance cutoffs (Westfall and Young 1993; Tusher et al 2001; Segal et al 2003; Storey and Tibshirani 2003). Frequently, however, signals in such data sets are too subtle to yield statistics that pass rigorous significance cutoffs.

One particularly straightforward method to reduce dimensionality is to select only those variables that meet criteria that are orthogonal to the property being investigated. For the purposes of this discussion, we define a ‘data type’ as a category of qualitative or quantitative information gathered from a biological sample. In this study, the data types include mRNA expression level measurements, genomic copy number measurements, and patient survival data associated with ovarian tumors. A ‘variable’ is defined as a single measurement of a multivariate data type. Variables would include mRNA measurements for individual genes or genomic copy number measurements for individual genomic regions.

Biological annotations can provide such a means of variable selection. By restricting a data set to only those variables whose annotations meet certain criteria, the dimensionality of the data set may be reduced such that multiple comparisons do not predominate. This concept, properly generalized, also supports integrated analysis across data types and across data sets. Given a series of tumor samples of known outcome, with experimental data comprising both DNA copy number and mRNA expression measurements, natural questions tend to span data types or require data annotation. Does genomic copy number directly account for some of the variation in gene expression across samples? Are the genes that map to loci that are frequently found to be of aberrant copy number more likely to show an association with outcome than other genes? Are genes that have functional annotations for processes involved in cancer (for example, adhesion, apoptosis, invasion, ...) more likely to be associated with tumor aggressiveness?

Questions such as these, which combine aspects of biology with aspects of statistics, tend to be very difficult for biological researchers themselves to answer during exploratory data analysis. Conversely, expert data analysts that have the skills required to answer such questions do not generally have the background to ask biologically motivated questions that might be stimulated by exploratory analysis. Since interpretation and exploration of data benefit from the background knowledge of the biologist, we have implemented a web-based system called Magellan that supports integrated analysis of complex, heterogeneous data sets. Magellan’s target audience is not experts in data analysis, but experimentalists that wish to explore their own data using established analytical methods while incorporating biologically meaningful annotations.

Here we present the Magellan system and its use in the analysis of combined comparative genomic hybridization (CGH), mRNA expression, and clinical data from 20 ovarian tumor samples (including 10 short survivors and 10 long survivors). Magellan supports univariate data analysis (with corrections for multiple comparisons), which identified one gene (spermidine/spermine N1-acetyltransferase) whose mRNA expression pattern correlated significantly with patient survival. Analyzing CGH data, we observed no single genomic locus whose gain or loss correlated significantly with patient survival, but a simple classification approach correctly predicted patient survival in 85% of cases under cross-validation. By making use of biological annotations including genomic mapping locations and functional gene annotations, more complex observations could be quantified: 1) DNA copy number and gene expression level are correlated genome-wide, on average; 2) genomic loci that were enriched for genes whose variation in expression correlated with survival exhibited copy number variation that correlated better with survival than all loci; and 3) genes that mapped to genomic loci that were frequently altered in copy number showed variation that correlated better with survival than all genes.

Magellan is part of the National Cancer Bioinformatics Grid (CaBIG) Project (http://cabig.nci.nih.gov/). Source code and documentation are available by email request. This system supports the use of open source analytical tools such that new methods of analysis can be rapidly prototyped and deployed. As part of the CaBIG project, instances of Magellan will be deployed at multiple institutions in support of cancer research across a wide community of experimentalists.

## Methods and Data

The following describes the analytical and computational methods in depth. The biological samples and experimental methods are presented briefly, since the experimental methods have been described in detail elsewhere.

### Experimental Samples and Biological Data

Twenty primary human ovarian tumor samples were analyzed in this study. Ten were tumors from patients that survived more than 7 years (*long* survivors), and 10 were from patients that survived less than 3 years (*short* survivors).

The tumors were analyzed by array CGH using experimental procedures previously described (Hodgson et al 2001; Snijders et al 2001). Briefly, DNA samples from the tumors and from normal tissues were labeled with CY3 and CY5, respectively, and hybridized to array comprised of replicate BAC clones distributed at ~ megabase intervals along the genome. Hybridized arrays were counterstained with DAPI to facilitate array element segmentation. CY3/CY5/DAPI images were analyzed to determine CY3/CY5 intensity ratios for each element in the array using custom software (Jain et al 2002). Measurements of individual array elements were discarded if the within-spot Cy3:Cy5 pixel intensity correlation was <0.81, or if the number of pixels per array element was <25 (the average spot size was 65 pixels) or if the CY3:CY5 ratio was >20% from the mean of 4 replicate measurements). This resulted in 2309 genomic loci after quality control and elimination of loci where more than 50% of samples had missing values. Note that missing values are typically caused by rejection of spots based on statistical concerns such as high replicate spot variance. Regions of the genome that have been deleted in the tumors should still be represented in the data set.

Expression data was collected as reported (Lancaster et al 2004), using the Affymetrix HuGeneFLGeneChip and Microarray Analysis Suite. This resulted in 7129 gene expression values for each sample.

### Data Annotation

As described above, one of the challenges posed by modern experimental methods is that even with the largest practical sample sizes, frequently the number of measurements vastly exceeds the number of samples. Another challenge is heterogeneous data types, where multiple experimental methods are used on a common set of samples. Magellan makes use of annotation information, which can be used to reduce dimensionality within a single data type, but which also provides a rich means for working across data types (and even across data sets).

Annotations are generally defined as textual or numeric information that describes the variables of a data type. Annotations within Magellan can be either *curated* or *derived.* Curated annotations can consist of numerical information such as genomic mapping data for genes, textual information derived from ontologies of gene function, and formal descriptions of regulatory networks (Ashburner et al 2000). Data and curated annotations are linked together through the use of ‘identifiers’, which are user-defined names such as Genbank or RefSeq IDs.

Derived annotations are *computed* from the experimental measurements that comprise a data set. These annotations are the results of a computation performed on the data and are, therefore, derived from that data. Derived annotations are typically quantitative and are linked to data directly rather than through identifiers. Direct linkage is required since individual identifiers (representing one gene on a microarray, for example) can be represented multiple times in a data set, with different quantitative measurements in each physical instance yielding different annotation values. For the analyses performed here, we used derived annotations of correlation to patient outcome for gene expression and frequency of alteration of genomic copy number.

### Magellan: A Web-Based System for Generalized Data Analysis

The primary aim of Magellan is to allow researchers to perform exploratory analyses of their data sets (hence the name Magellan). Biological data analysis has been the subject of intense research in many groups, and there are a number of systems available that are geared toward a similar user community. These range from single-use packages (such as clustering) to integrated packages for performing multiple types of analysis (such as univariate analyses and classification for multiple data types). Examples of the former include methods such as SAM and PAM, which address permutation-corrected statistics and pattern classification, respectively (Tusher et al 2001; Tibshirani et al 2002), and Cluster and Tree-View (Eisen), which address clustering. Magellan differs from these in that it is a platform for offering multiple types of analysis. Examples of the latter include MeV (TIGR), caWorkBench (Columbia Genome Center), GeneCluster (Broad Institute), Gene-Sifter (VizX Labs LLC), and mAdb (NCI).

In terms of functionality, Magellan’s primary distinguishing characteristics are its generality and its use of biological annotation information as a means of constraining analyses to variable subsets. Magellan is general in two respects. First, the internal schema for storing information supports any type of data that can exist in a table (either numerical or textual). Second, Magellan does not impose complex format requirements on data, which is frequently a hurdle in making use of other systems, where local procedures for data preparation may be at variance with expectations and requirements for data formatting. Magellan currently allows users to define the content and location of relevant information in a data file prior to storage of that information in the database. As part of the caBIG project, Magellan will be extended to accept several standard file formats.

**Figure 1:**
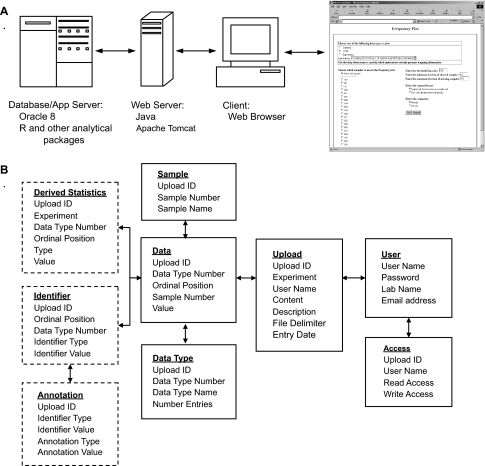
Magellan architecture and database schema.
System architecture, including the applications running on the various system components. An example web page is also shown.Database table structure. Tables used to store annotation information are bordered with dashed lines.

**Figure 2:**
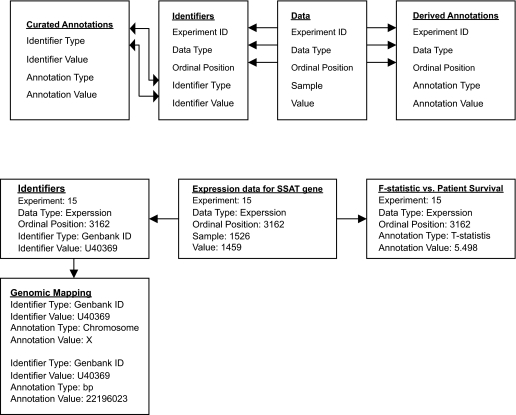
Linkage of data to curated and derived annotation information. Variables of a data type (such as genes or genomic loci) are referenced by their ordinal position. The ordinal position can be directly linked to derived statistics (such as t-test against patient survival) or to named identifiers (such as a Genbank IDs). Identifiers are then used to link data to curated annotations (such as genomic mapping information).

Stored data and annotations are treated within Magellan as abstract entities, such that arbitrary, user defined types of information can be stored. This abstract approach to data and annotation representation is similar to the EAV (Entity-Attribute-Value) approach for modeling heterogeneous data, which allows a flexible means of storing information without limits on the number and type of attributes per entity (Nadkarni et al 1999). The database schema used to store data and annotations is graphically represented in Figure 1. Using this approach, experimental data may be any number of multidimensional vectors of information that belong to a sample. Likewise, annotations are abstractly defined as textual or quantitative information that describes one or more variables of a given data type, such as genomic position or biological pathway information for a particular gene in an mRNA expression data set (Figure 2). Thus, an arbitrary number of user defined data and annotation types can be loaded and stored in the system, allowing very broad utility.

Magellan’s annotation representation was designed with flexibility in mind, since much of the existing annotation information is informal and derived from varied sources. There do exist, however, many ‘standardized’ sources of annotations that have been curated by third parties such as the Gene Ontology Consortium. In the current version of Magellan, these annotations may be used by direct upload of the information, as with any other source, standard or not. As a convenience feature, direct retrieval of annotations from within caBIG enabled sources is part of the planned public release of Magellan. Users will be able to use Magellan to retrieve and utilize the standardized annotations curated as part of the caBIG project.

Magellan’s architecture differs from most of the systems mentioned above in that it is a thin-client application, where the only requirement on a user is that they have a Web browser and an Internet connection. Magellan is based on an open architecture where integration of new analytical methods is straightforward. The intention is that a limited number of Magellan instances will be deployed in order to cover a large number of users, and new analytical methods will be interfaced as needed in order to support the user base. Access to all information stored in Magellan is password protected, and access rights can be conferred upon selected individual users or made public, based on the preferences of the owner of the data.

The web pages that provide the user interface to the Magellan system were developed using Java Server Pages technology. The use of Java allows developers to add functionality and deploy new algorithms to Magellan relatively easily, since much of the logic is contained in compiled Java classes that can be accessed via the documented API and modified via inheritance. Magellan is designed to run on the Windows 2000 operating system, using the Apache Tomcat JSP servlet container, the Ant development tool, and Java 1.2. All information is currently stored using Oracle 8.1 database software, although migration to an open source database application such as MySQL is possible in future releases. During development of Magellan, an attempt has been made to utilize standardized database interaction methods and procedures in order to facilitate such a port in the future. Analytical methods are currently deployed as compiled C executables or using the open source statistical package R, although any software that can be accessed via command line interfaces or scripts can potentially be interfaced to Magellan.

Additional details about the current version of Magellan (source code and documentation) are available by email request. An open-access version will be released as part of the caBIG deployment. A link to an instance of Magellan, pre-loaded with the data presented in this paper can be found at www.jainlab.org/publications.html. Note that while Magellan restricts user access to data owned by the specific user, network communications are not encrypted. Further, since the data model allows any type of data to be stored, it is left to the users to avoid storing sensitive data such as personally identifiable information about patients. All data reported here and present on the publicly available system has been de-identified.

## Results

As described above, CGH and mRNA expression data were obtained from twenty ovarian tumors, comprising ten long and ten short survivors. We used Magellan to explore the relationship between these two types of genomic data, and between the genomic data and accompanying clinical information. We performed standard analyses, which made use of a single data type at a time, with no use of annotation information. We also performed multiple analyses that required annotation information, multiple data types, or both.

### Single-Mode Analysis

**Figure 3:**
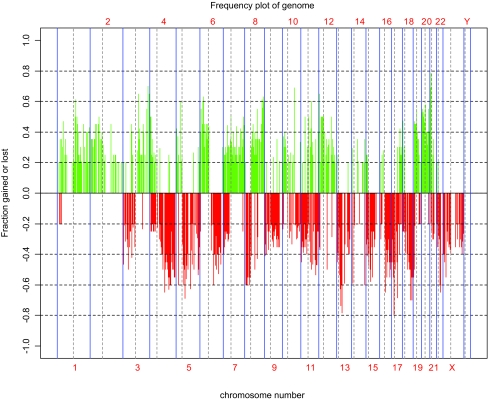
Graphical representation of the frequency of chromosomal gains/losses in 20 ovarian tumors. The ratio of genomic copy number in tumor vs. normal was log_2_ transformed and plotted against genomic position. Only those loci for which at least 20% of samples were aberrant at a log threshold of 0.25 were plotted

Figure 3 shows the frequency of genomic copy number alteration for the twenty samples. Gains and losses previously shown to be common in ovarian tumors using chromosomal CGH (Shayesteh et al 1999) are present. The resolution of array-based CGH further sharpens the structure of the abnormalities.

Correlation analysis was used to determine the relationship between genomic and clinical data, that is whether the behavior of any single gene or genomic locus significantly correlated with patient survival class by t-test. The significance threshold for the t-statistic was determined by a permutation-based approach, as described previously (Jain et al 2001) using the 95^th^ percentile of the max permutation distribution as a significance cutoff. We found a significant correlation between the expression of the spermidine/spermine N1-acetyltransferase (SSAT) gene and patient survival at p<0.05. This is a conservative approach, since the null distribution is calculated from the maximum observed t-statistic from each round of permutation. The SSAT gene has been previously observed to be elevated in human prostate cancer, where it may have a role in maintaining polyamine homeostasis (Bettuzzi et al 2000). While the biological and clinical interpretation of the correlation between SSAT and patient survival in ovarian cancer is unclear at this point, the result demonstrates use of Magellan to identify a correlation between gene expression and clinical outcome under a stringent test of significance.

**Figure 4:**
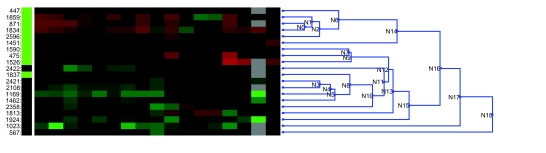
Hierarchical clustering of samples based on loci whose DNA copy number correlated strongly with outcome. The t-statistic of copy number gain/loss for each genomic locus was used to select those loci that correlated strongly with outcome, and these restricted loci were used to cluster ovarian tumor samples. Survival – Green: long term survivors, Black: short term survivors. CGH Data – Green: gain of copy number, red: loss of copy number.

An identical analysis of the CGH data revealed no single locus with significant association to clinical outcome. However, clustering samples using CGH loci whose copy number correlated with outcome showed a good segregation of samples based on class, suggesting possible success of outcome classification by CGH data (Figure 4). K-nearest neighbors classification with variable selection (Olshen and Jain 2002) yielded reasonable classification performance under cross-validation. Variable selection was performed for each round of cross validation, using the t-statistic vs. outcome to select the top loci for each sample holdout. Using the Euclidean distance metric, we consistently observed a better than random fraction of correctly classified tumors using a number of different values for parameters such as the number of holdouts, number of loci, and number of neighbors (Table 1a, Table 1b). Due to the very small sample size, cross-validation estimates of classification success were better for leave-one-out testing than for tests run with larger holdout sets. For the single-holdout cases, classification performance was 80 to 85% (Table 1a). Table 1b shows the effect of varying the number of selected loci under different values of K, using a single holdout. With a very small number of selected variables, performance was poor, but there was a broad peak of performance from 25 to 100 variables, centered at 50. Classifiers based on mRNA expression data underperformed classifiers based on CGH data, despite the converse result in the univariate analyses.

Given the very small number of samples, these results are encouraging to a degree, but require prospective validation on larger sample sets. The signal within the data is not overwhelmingly strong, and the single-mode analyses do not reveal compelling results with respect to patient outcome.

### Annotation Based Correlation Methods

The preceding analyses were performed independently on the mRNA expression and CGH data sets. In order to *relate* the two data sets, we relied on annotations associated with the data. In particular, we used genomic mapping data to relate genes to genomic loci (i.e. to identify corresponding variables in the two multivariate data types). We present two types of analysis: 1) where a statistical question relies directly upon annotation information, and 2) where a statistical computation is used as an annotation of genes in order to constrain a question about genomic loci. The term ‘projection’ is used to describe the process of finding corresponding variables between two data types by examining the relationship between the annotations associated with those variables.

*Gene Dosage Effect:* The presence of frequent alterations in copy number across the genome in this collection of ovarian tumors (Figure 3) suggests that many genes could potentially be affected by gains or losses of genomic DNA. In order to determine the relationship between CGH and expression data (whether the sample-to-sample copy number of any genomic locus was correlated with the mRNA expression of any gene), we performed a correlation analysis similar to that between genomic data and clinical data. Correlation coefficients were calculated for each pairwise combination of measurements for loci and genes, and significance was determined by permutation analysis. No such correlations exceeded the 95th percentile cutoff of the permutation analysis, indicating the inherent difficulty of finding relationships between high-dimensional data types with a relatively small number of samples.

We considered a more direct relationship between gene copy number and gene expression by incorporating biological annotations that used the genomic mapping of the BACs of the CGH array and the genes on the Affymetrix chip. The annotations were used to create bins of genes and loci based on common values. In this analysis, the genome was divided into 100 bins of equal length. Each bin of a two-dimensional matrix contained the genes (x-axis) and loci (y-axis) that mapped to those genomic regions indicated on the respective axes. The color of each bin was the average of all cross correlations of the DNA copy number for each genomic locus vs. the mRNA expression level of each gene within the bin.

**Figure 5:**
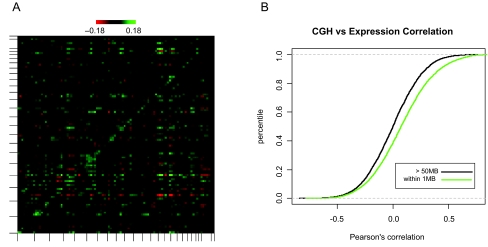
Correlation of CGH and mRNA expression data, binned by genomic position. A: All by all Pearson's correlation of CGH and expression data, binned by genomic position as described in the text. B: Cumulative distributions of gene/locus correlations for pairs that are within 1 Mb of each other (green) or at least 50Mb away from each other (black).

Figure 5a shows a graphical depiction of the result computed and rendered from Magellan. An area of higher than background correlations is apparent along the diagonal of the graph, suggesting that expression of genes correlated with the amplification/deletion of loci that map nearby in the genome. The significance of off diagonal correlation values is uncertain, but the correlation values for genes and loci that map to within 1Mb is significantly different from the correlation values for genes and loci that are separated by at least 50Mb (p << 0.001 by t-test). This is evidenced by the right-shift of the cumulative histogram of correlation values in the close-mapping case (Figure 5b).

Genome-wide, on average, amplification of a genomic region tends to up-regulate the expression of genes in that region while genomic deletion tends to down-regulate gene expression, a result that has also been observed in breast cancer (Pollack et al 2002). The result confirms expectations, but there are two aspects of this analysis with respect to the Magellan platform that are important. First, the explicit use of annotation information (genomic mapping data) was required to make the correspondence between data types. Second, integration of the annotations with the data for the analysis was accomplished using a procedure easily accessible to a naïve user.

*Gene Annotations:* Gene Ontology annotations were available for most of the genes represented in the data set. These were loaded in Magellan as cu-rated annotations of the expression data type. As a demonstration of textual annotation use, we selected 394 genes with any of 13 cell cycle related gene annotations (features such as cell cycle, regulation of cell cycle, ...). We compared the distribution of gene correlations with survival from all genes versus those of the cell cycle, observing a rightward shift of the latter set. The shift was not quite significant (p = 0.07 by Wilcoxon rank sum) and are presented here as an illustration, but the following results using *derived* annotations exhibited significant enrichment of extreme statistics.

*Derived Annotations:* In addition to curated annotations such as genomic position, data can be selected based upon quantitative annotations that are derived from the data itself (such as correlation with a clinical outcome) prior to analysis. This process can be useful in moving among data types within an experiment as well as moving between different experiments.

**Figure 6:**
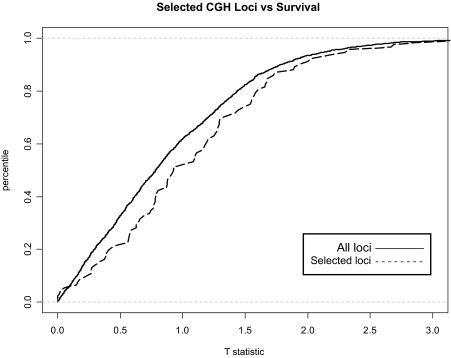
Projection of variables among two data types: from measurements of mRNA expression to DNA copy number by CGH. Cumulative distribution of t-statistics of CGH loci vs. survival for all loci (solid line) or loci that are within 1MB of one of the top 1% of genes that correlated with survival (dashed line). The distribution of statistics from subselected loci is significantly shifted from unselected loci (p < 0.05 by Wilcoxon rank-sum test).

We computed the t-test for the mRNA expression data with respect to patient survival for each gene and selected the option within Magellan to store the values as a derived annotation for the expression data type. The expression data set was then sub-selected such that only those genes with statistic values in the top 1% were considered. Using the genomic mapping of the genes, those loci in the CGH data set that were in close physical proximity to the selected genes were selected. The cumulative histograms from the distribution of t-statistics for the 95 selected loci and the corresponding distribution for all loci are shown in Figure 6. The cumulative histogram for the selected loci is shifted to the right of that of the unselected loci, indicating that genomic aberrations that are located near genes whose expression correlates with outcome exhibit copy number variation that is more strongly correlated with outcome (p < 0.05 by Wilcoxon rank-sum test).

In data sets with limited numbers of samples, such as the one presented here, it is often easier to observe significant differences in distributions than in individual genes or loci. In the example just presented, we projected variables from the gene expression data type to the gene copy number data type, but we made use of outcome information in the process. Since we have shown a direct relationship between measurements of genome copy number and expression, the effect may be a simple manifestation of the same process.

**Figure 7:**
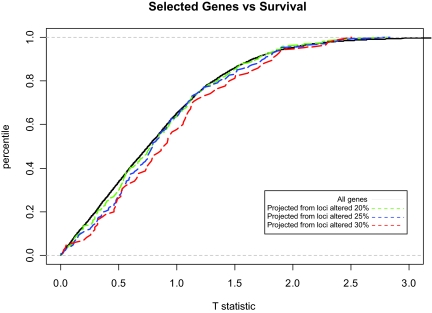
Projection of variables among two data types: from measurements of DNA copy number by CGH to mRNA expression. Cumulative distributions of t-statistics of mRNA expression vs. survival for all genes (solid black line) or genes that are within 1MB of CGH loci whose log2 deviation from normal is at least 0.5 at a frequency of at least 20% (dashed green line), 25% (dashed blue line), or 30% (dashed red line) of samples. The difference between the distributions for all genes compared with those genes that map to loci that are altered in 30% or more samples is significant (p < 0.05 by Wilcoxon rank-sum test).

However, since genome copy number has an expected normal value (a relative log ratio of 0 compared with normal), we can identify genomic loci that are involved with the disease process *without* reference to outcome. We identified genomic loci that met progressively more stringent tests of frequency of aberration and considered the behavior of the genes that mapped close by with respect to patient outcome. Figure 7 shows cumulative histograms of the t-statistics of gene expression for multiple subsets of genes. The leftmost distribution was from all genes. Each succeeding distribution was computed from progressively more stringent criteria. The shift in distributions is monotonic, and the difference between the distributions for all genes compared with those genes that map to loci that are most frequently altered is significant (p < 0.05 by Wilcoxon rank-sum test).

## Conclusions

We have developed an analytical system capable of performing complex analyses on high-dimensional data and annotations. We demonstrated Magellan’s application on a specific genomic data set containing CGH, mRNA gene expression, and clinical outcome data from ovarian tumors. Using this system, we were able to find a significant correlation between expression of the SSAT gene and patient survival, and we are able to build a CGH based classifier that correctly predicts survival 85% of the time in cross validation. It is important to note that these results are derived from a small data set comprising a selected group of patients, and any conclusions need to be reproduced on larger data sets.

By making use of curated annotations, stronger conclusions about the relationship between genome copy number and gene expression could be made. In particular, on average, genome-wide, there is a significant component of gene expression variation that is explained by changes in genomic copy number. Using combinations of curated and derived annotations, we showed enrichment for relationship to survival in moving from gene expression data to genome copy number data and *vice versa.*

The analyses are possible in the context of a thin-client architecture that is accessible to the community of experimental biologists. End users access Magellan through a web browser, providing a very low barrier for use of the system; there is no software to install on the client-side. Magellan includes web-based documentation to assist users in the uploading and analysis of data and annotations. The system architecture is built around central web, database, and application servers that store all data and annotations in a relational database. Because data and annotations are represented abstractly, users are able to perform a limitless variety of annotation-based selections on virtually any type of data prior to analysis.

Magellan is part of the National Cancer Bioinformatics Grid (CaBIG) Project. Currently, the upload, storage, and analysis functions described in this manuscript are functionally mature. Within the next year, connectivity to the data and annotation stores at the NCI will be achieved. In addition, several open source statistical tools will be incorporated into Magellan, particularly the R based tools of the Bioconductor project such as the aCGH package for the analysis of CGH data. The Magellan source code and documentation are available by email request. A link to an instance of Magellan preloaded with the data described in this paper is also accessible online at www.jainlab.org/publications.html. As part of the CaBIG project, instances of Magellan will be deployed at multiple institutions in support of cancer research across a wide community of experimentalists.

Magellan’s source code and documentation will become publicly available within the caBIG web-based CVS system. The first public release, scheduled for early 2006, will include the functions described here and will also include improvements in interface functionality, online documentation, and standardized annotation retrieval. These aspects are being developed in collaboration with formal adopters in the caBIG community and represent an ongoing collaboration among developers and users of Magellan.

## Figures and Tables

**Table 1:** Fraction of tumors correctly classified using CGH data under cross validation. Tumors were classified using KNN with different numbers of holdouts, different numbers of genomic loci, and different values of K (the number of neighbors). The fraction of tumors that were correctly classified for each set of parameters is reported. A. Fraction of tumors correctly classified using 50 genomic loci but varying the number of holdouts and the value of K. B. Fraction of tumors correctly classified using one holdout but varying the number of genomic loci and the value of K.

	**K**				

		**1**	**3**	**5**	**7**
	**1**	.85	.85	.80	.80
Holdouts	**2**	.75	.70	.65	.50
	**3**	.80	.75	.70	.60
	**4**	.60	.80	.70	.70
	**5**	.55	.70	.55	.50

	**K**				

		**1**	**3**	**5**	**7**
	**5**	.50	.45	.40	.65
Loci	**10**	.70	.50	.55	.60
	**25**	.70	.80	.70	.60
	**50**	.85	.85	.80	.80
	**100**	.70	.75	.65	.55
